# Acute Binge Drinking Increases Serum Endotoxin and Bacterial DNA Levels in Healthy Individuals

**DOI:** 10.1371/journal.pone.0096864

**Published:** 2014-05-14

**Authors:** Shashi Bala, Miguel Marcos, Arijeet Gattu, Donna Catalano, Gyongyi Szabo

**Affiliations:** Department of Medicine, University of Massachusetts Medical School, Worcester, Massachusetts, United States of America; University of Louisville School of Medicine, United States of America

## Abstract

Binge drinking, the most common form of alcohol consumption, is associated with increased mortality and morbidity; yet, its biological consequences are poorly defined. Previous studies demonstrated that chronic alcohol use results in increased gut permeability and increased serum endotoxin levels that contribute to many of the biological effects of chronic alcohol, including alcoholic liver disease. In this study, we evaluated the effects of acute binge drinking in healthy adults on serum endotoxin levels. We found that acute alcohol binge resulted in a rapid increase in serum endotoxin and 16S rDNA, a marker of bacterial translocation from the gut. Compared to men, women had higher blood alcohol and circulating endotoxin levels. In addition, alcohol binge caused a prolonged increase in acute phase protein levels in the systemic circulation. The biological significance of the in vivo endotoxin elevation was underscored by increased levels of inflammatory cytokines, TNFα and IL-6, and chemokine, MCP-1, measured in total blood after in vitro lipopolysaccharide stimulation. Our findings indicate that even a single alcohol binge results in increased serum endotoxin levels likely due to translocation of gut bacterial products and disturbs innate immune responses that can contribute to the deleterious effects of binge drinking.

## Introduction

Alcohol binge drinking defined as more than 4 drinks consumed is the most frequent form of alcohol consumption worldwide. This drinking pattern is popular among underage drinkers and young adults leading to increased mortality and morbidity [1]–[3]. While the behavioral consequences of binge drinking are well characterized, little is known about its systemic effects on various organs and on immune responses. On immune cells, acute alcohol intake has immunosuppressive and anti-inflammatory effects, it reduces antiviral immunity and in the liver it causes liver injury and steatosis [4], [5].

Animal models of chronic alcohol administration and human studies indicate that alcoholic liver disease is associated with increased endotoxin (lipopolysaccharide; LPS) levels in the portal and systemic circulation and that endotoxin contributes to alcoholic steatohepatitis [3], [6], however, interplay between endotoxin and binge drinking is not well understood. LPS is a Toll-like Receptor 4 ligand that triggers inflammatory cytokine activation in immune cells and has a broad range of effects on various cell types in different organs [6]. Increased serum LPS is the result of increased gut permeability and it is associated with a low-grade inflammation in various conditions such as alcoholic hepatitis, cirrhosis, HIV and HCV infection [7]. Impaired gut barrier function, bacterial translocation and alterations in the gut microbiome are found in animal models of chronic alcohol consumption [8], [9]. An alteration in colonic microbiome was also reported in chronic alcoholics [10]. In this study, we tested the effects of acute binge drinking on serum endotoxin and bacterial 16S rDNA in normal human adults.

## Materials and Methods

### Human studies

Healthy individuals (11 males and 14 females, age range 21–56) with no history of alcohol use disorder were enrolled in the study after informed consent was obtained. All participants signed written consent and the study was approved by Institutional Review Board for the Protection of Human subjects in research at the University of Massachusetts Medical School. Written consent files are kept in locked cabinets and samples were de-identified. Alcohol use habits were determined by a questionnaire that incorporated the Alcohol Use Disorders Identification Test and CAGE tests as described [11]. To qualify for the study, males had alcohol use of fewer than 12 drinks/wk, females fewer than 9 drinks/wk, and all abstained from alcohol for at least 48 hours before participation [11]. Alcohol was given @ 2 ml vodka 40% v/v ethanol/kg body weight in a total volume of 300 ml orange/strawberry juice in the Clinical Research Center (CRC). Blood was drawn at baseline and after every 30 minutes for the first 4 hours and again after 24 hours post alcohol consumption in serum separating tubes (BD Biosciences) as indicated in figures. Average body mass index (BMI) ± standard error of men and women was 29.3±1.1 and 28.8±1.38 respectively. For control subjects, blood samples were collected from alcohol-abstinent individuals (age- and gender-matched) receiving equal volume of orange juice.

### Alcohol concentration and Endotoxin Assay

The amount of alcohol in the serum samples was measured by an Analox Alcohol Analyzer. Serum endotoxin was measured using Endotoxin kit (Capecod Inc, USA) as per manufacturer instructions.

### Measurement of LBP and sCD14

LBP and sCD14 were measured from the serum using ELISA kits from Lonza (Lonza, USA).

### Bacterial DNA isolation and identification

DNA was isolated from equal volume of serum samples using QIAamp mini kit (Qiagen, USA) in endotoxin free conditions. PCR reaction was performed with universal 16S primer [12], and the product was analyzed on 1% agarose gel. *Escherichia coli DNA* was used as a positive control and “reaction mix alone” was used as a negative control to avoid any false positive amplification. PCR was performed using primer sequence, forward 5′-TCCTACGGGAGGCAGCAGT-3′ and the reverse 5′-GACTACCAGGGTATCTAATCCTGTT-3′, and an amplicon of 466 bp was detected. The fold change was calculated compared to baseline samples of the same individual. Experiment was performed in a bacterial free chamber.

### LPS treatment of whole blood samples

For blood cytokines, whole blood was diluted 1∶1 with warm RPMI in a polypropylene tube with the cap loose. Samples were either stimulated or not with LPS (100 pg/ml), then placed on a rocker platform in a CO2 chamber for 18 h. The next day tubes were centrifuged at 3000RPM for 10 minutes and clear plasma was collected and stored at −80°C for cytokine measurements.

### Measurement of cytokines

Plasma collected from LPS treated whole blood samples was used to measure TNFα, IL-6 and MCP1 using ELISA kits (Thermo Scientific, USA [TNFα] and BD Biosciences, USA [IL-6 and MCP1]).

### Statistical analysis

Depending upon the data distribution, statistical analysis was performed either with Mann-Whitney *U* test or Student's two-tailed test.

## Results

### Increased blood alcohol content and serum endotoxin after an acute binge drink in healthy humans

**Figure 1:**
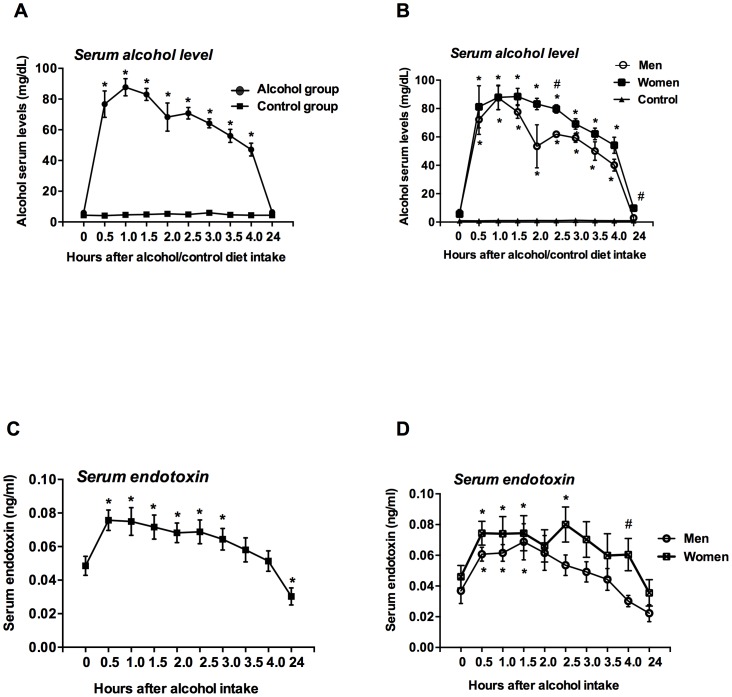
Induction of blood alcohol content and serum endotoxin after an acute binge drink in healthy humans. A–D. Serum alcohol (A and B, n = 8) and endotoxin (C and D, n = 16) levels were measured as described in methods. The data for serum alcohol (B) and endotoxin (D) was presented separately for men and women. Data is presented as mean ± S.E., and depending upon the data distribution, statistical analysis was performed either with Mann-Whitney *U* test or Student's two-tailed test. *p<0.05. *represents comparison between baseline and treatment groups. # represents comparison between men and women.

Alcohol intake increases blood alcohol level (BAL) in the circulation. In normal volunteers, acute alcohol binge resulted in maximum BAL at 60 minutes after alcohol consumption with a gradual decline thereafter (Fig. 1A). Compared to men, women showed a slower decrease in BAL and even 24 h after the alcohol binge BALs were higher in women than that in men (Fig. 1B). There was no detectable blood alcohol level (BAL) before alcohol intake in any of the individuals. Parallel to BAL levels, serum endotoxin levels rapidly increased by 30 minutes, remained elevated for 3 hours and returned to lower than baseline levels by 24 hours after alcohol intake (Fig. 1C). Serum endotoxin levels were also higher in women after alcohol intake and a significant difference in endotoxin level was observed at 4 h between men and women (Fig. 1D). Blood alcohol and serum endotoxin levels remained constant in control individuals who received no alcohol.

### Induction of acute phase proteins after an acute binge drink

**Figure 2:**
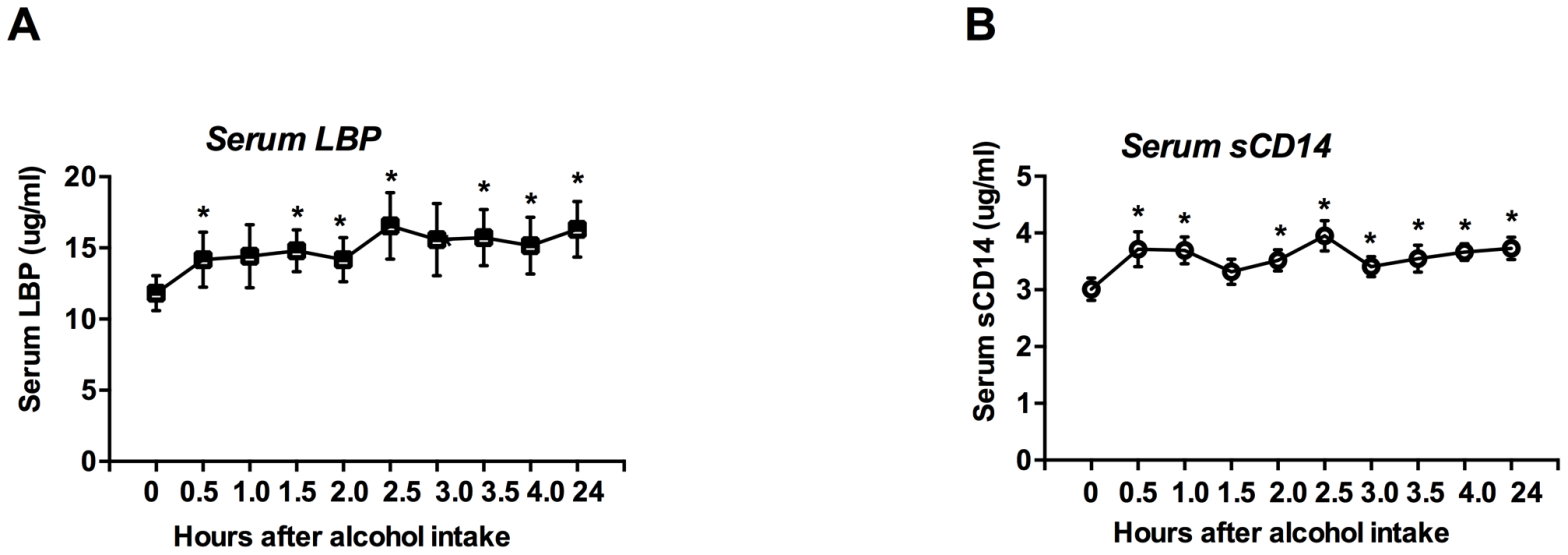
Induction of acute phase proteins after an acute binge drink in healthy humans. A–B. Serum LBP (A, n = 21) and serum sCD14 (B, n = 21) were measured as described in methods. Data is presented as mean ± S.E., and depending upon the data distribution, statistical analysis was performed either with Mann-Whitney *U* test or Student's two-tailed test. *p<0.05.

Circulating endotoxin induces elevations in acute phase proteins [13]. We found that acute alcohol consumption also induced rapid and prolonged increases in serum lipoprotein binding protein (LBP) (Fig. 2A) and soluble CD14 (sCD14) (Fig. 2B) while there was no change in controls who received no alcohol (data not shown).

### Induction of bacterial DNA after an acute binge drink in healthy humans

**Figure 3:**
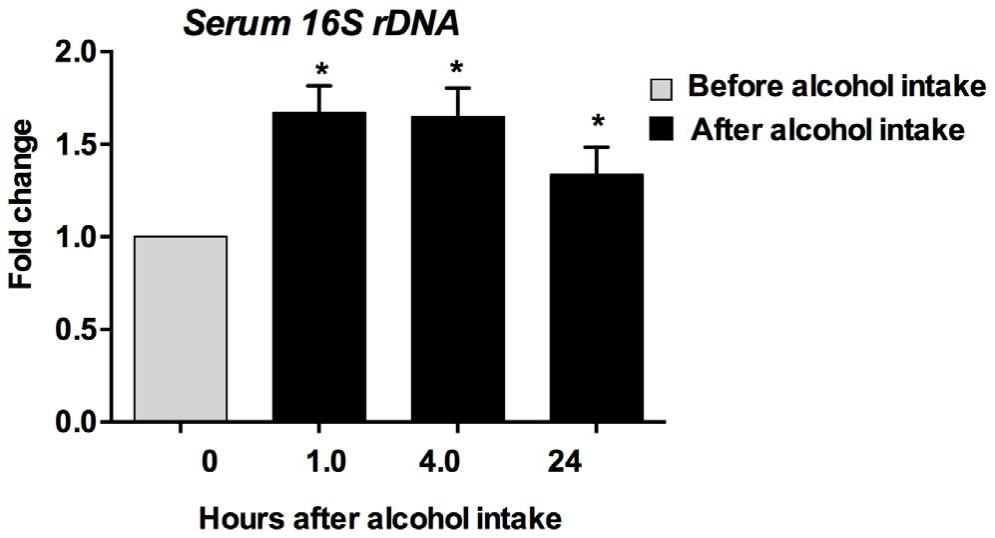
Induction of bacterial DNA after an acute binge drink in healthy humans. DNA was isolated from the serum (n = 22) using QIAamp mini kit (Qiagen) and 16S rDNA was amplified using 16S universal primers. The fold change was calculated compared to baseline samples of the same individual. Data is presented as mean ± S.E. *p<0.05.

LPS is a component of Gram-negative bacteria and increased serum LPS is thought to be a result of microbial translocation from the gut [14]. Because LPS represents only some enteric bacterial microbiota (Gram negative) we further determined the levels of the well-conserved 16S rRNA subunit (16S rDNA), which is common to most bacteria. We found a significant increase in serum 16S bacterial rDNA levels at 1, 4 and even 24 hours after binge drinking (Fig. 3) and not in controls (data not shown).

### “Physiological” dose of LPS induces immune response in whole blood of normal individuals

**Figure 4:**
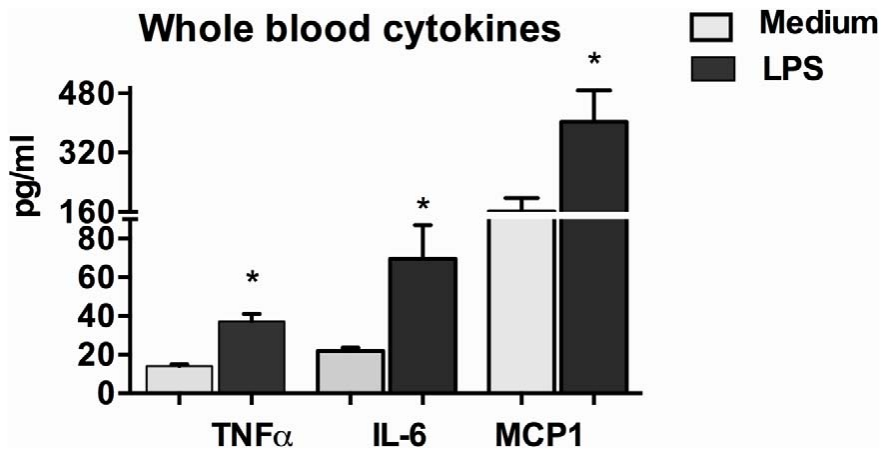
Physiological dose of LPS induces immune response in whole blood plasma of normal individuals. The amount of TNFα, IL-6 and MCP1 was measured by ELISA from the whole blood plasma collected after 18 h of LPS (100 pg/ml) treatment (n = 4). Data is presented as mean ± S.E. *p<0.05.

To evaluate the biological significance of the serum endotoxin increase found in vivo after acute alcohol binge, we evaluated the effect of the biologically comparable concentrations of LPS in whole blood of normal volunteers. We found that the concentrations of endotoxin observed in the serum after acute binge had significant biological activity. In vitro treatment of whole blood samples with a “physiological” dose of LPS (100 pg/ml for 18 h) resulted in a significant induction of inflammatory cytokines, tumor necrosis factorα (TNFα), IL-6 and MCP1 in normal subjects (Fig. 4).

## Discussion

Our novel data demonstrate that a single alcohol binge in normal subjects resulted in a rapid and transient increase in serum endotoxin levels and that even this modest increase in serum LPS had substantial biological effects in modulating TNFα, IL-6 and MCP1. Endotoxin, LPS, is a potent trigger of the inflammatory cascade via activation of the TLR4 complex [6]. Recent studies in mice indicate that in addition to its effects on innate immunity, LPS administration also contributes to alcohol dependence and promotes prolonged increase of alcohol intake in mice [15]. Mutant mice lacking genes related to immune function exhibit decreased alcohol consumption indicating immune signaling promotes alcohol consumption [16]. Thus, it is tempting to speculate that LPS increase in the systemic circulation after an acute alcohol binge could promote the desire for alcohol consumption. This speculation merits further investigation. Consistent with our data, acute ethanol exposure in rodents (rats) is shown to increase circulating endotoxin levels [17]. The systemic biologic activity of the increased LPS after alcohol binge was associated with increased serum LBP and soluble CD14 levels. LBP and sCD14 are both LPS-inducible protective molecules and are considered as “acute phase reactants” [18]. Increases in LBP and sCD14 have the biologic function of binding circulating LPS thereby preventing activation of the TLR4 complex [18]. In chronic alcohol use activation of the inflammatory cascade is a major component of organ damage in the brain and liver. Alcohol binge can cause altered immune functions that can also contribute to immunosuppression and reduced immune-mediated host defense to pathogens [3]. Despite similar BMI between men and women in our study, women showed higher levels of blood alcohol and circulating endotoxin levels. This could be due to the fact that the volume of distribution in women is less than that in men and gender differences in the effects of binge drinking deserve additional investigation.

Our study also demonstrated the elevation of other bacterial products such as 16S rDNA levels after acute binge drinking indicating transient gut-derived microbial translocation as a likely mechanism for the serum LPS increase. It is noteworthy that increase in circulating bacterial components after acute binge not only included Gram-negative bacteria (LPS) but extended to other enteric bacterial elements such as 16S rDNA. The significance of increased bacterial DNA in alcohol binge model deserves further investigation as it may represent a danger signal that is different from LPS. The persistent increase in bacterial components including 16S rDNA after repeated binge drinking might result in immune activation thereby resulting in inflammation and initiation of alcoholic liver disease. Increase in bacterial translocation and 16S rRNA was found in chronic alcohol mouse model [19]. In other pathological conditions such as in HIV infection, plasma levels of bacterial DNA have been shown to correlate with immune activation [20] and translocation of microbial elements is also observed in inflammatory bowel disease [21].

It is possible that LPS along with bacterial DNA activates the immune system during binge drinking. The pro-inflammatory effects of chronic alcohol are associated with increased serum LPS and this is thought to be due to alcohol-induced impairment of the gut barrier function [8]. To our best knowledge, we show for the first time that acute alcohol binge causes 16S rDNA translocation (conserved bacterial genetic component) into the systemic circulation in otherwise healthy adults. The mechanism of alcohol-induced gut barrier dysfunction is likely multifactorial. Decrease in tight junction proteins, occludin and zonulin-1 were found after chronic alcohol use and this was mechanistically linked to increased expression of miRNA-212 in mice [22]. Expression levels of defensins that have protective effects in the gut are also decreased in chronic alcohol feeding in mice although the effects of acute alcohol binge on these proteins are yet to be explored [19].

In summary, our study demonstrates that acute alcohol binge increased serum levels of bacterial products (endotoxin and bacterial 16S rDNA) which might contribute to innate immune responses and potentially to the behavioral effects of alcohol binge drinking. These results suggest that acute binge drinking has significant adverse health effects even in healthy individuals.

## References

[1] NIAAA website. Available: http://pubs.niaaa.nih.gov/publications/aa68/aa68.htm. Accessed 2014 Jan 10.

[2] Mathurin P, Deltenre P (2009) Effect of binge drinking on the liver: An alarming public health issue? Gut 58: 613–617. 19174416 10.1136/gut.2007.145573

[3] Shukla SD, Pruett SB, Szabo G, Arteel GE (2013) Binge ethanol and liver: New molecular developments. Alcohol Clin Exp Res 37: 550–557. 23347137 10.1111/acer.12011 PMC4831914

[4] Bala S, Tang A, Catalano D, Petrasek J, Taha O, et al (2012) Induction of bcl-3 by acute binge alcohol results in toll-like receptor 4/LPS tolerance. J Leukoc Biol 92: 611–620. 22782967 10.1189/jlb.0112050 PMC3427604

[5] Massey VL, Arteel GE (2012) Acute alcohol-induced liver injury. Front Physiol 3: 193. 22701432 10.3389/fphys.2012.00193 PMC3372892

[6] Szabo G, Bala S, Petrasek J, Gattu A (2010) Gut-liver axis and sensing microbes. Dig Dis 28: 737–744. 21525758 10.1159/000324281 PMC3211517

[7] Szabo G, Wands JR, Eken A, Osna NA, Weinman SA, et al (2010) Alcohol and hepatitis C virus—interactions in immune dysfunctions and liver damage. Alcohol Clin Exp Res 34: 1675–1686. 20608905 10.1111/j.1530-0277.2010.01255.x PMC3253556

[8] Keshavarzian A, Farhadi A, Forsyth CB, Rangan J, Jakate S, et al (2009) Evidence that chronic alcohol exposure promotes intestinal oxidative stress, intestinal hyperpermeability and endotoxemia prior to development of alcoholic steatohepatitis in rats. J Hepatol 50: 538–547. 19155080 10.1016/j.jhep.2008.10.028 PMC2680133

[9] Hartmann P, Chen WC, Schnabl B (2012) The intestinal microbiome and the leaky gut as therapeutic targets in alcoholic liver disease. Front Physiol 3: 402. 23087650 10.3389/fphys.2012.00402 PMC3468817

[10] Mutlu EA, Gillevet PM, Rangwala H, Sikaroodi M, Naqvi A, et al (2012) Colonic microbiome is altered in alcoholism. Am J Physiol Gastrointest Liver Physiol 302: G966–78. 22241860 10.1152/ajpgi.00380.2011 PMC3362077

[11] Norkina O, Dolganiuc A, Catalano D, Kodys K, Mandrekar P, et al (2008) Acute alcohol intake induces SOCS1 and SOCS3 and inhibits cytokine-induced STAT1 and STAT3 signaling in human monocytes. Alcohol Clin Exp Res 32: 1565–1573. 18616672 10.1111/j.1530-0277.2008.00726.x PMC4116614

[12] Horz HP, Vianna ME, Gomes BP, Conrads G (2005) Evaluation of universal probes and primer sets for assessing total bacterial load in clinical samples: General implications and practical use in endodontic antimicrobial therapy. J Clin Microbiol 43: 5332–5337. 16208011 10.1128/JCM.43.10.5332-5337.2005 PMC1248440

[13] Levy O, Teixeira-Pinto A, White ML, Carroll SF, Lehmann L, et al (2003) Endotoxemia and elevation of lipopolysaccharide-binding protein after hematopoietic stem cell transplantation. Pediatr Infect Dis J 22: 978–981. 14614371 10.1097/01.inf.0000095196.19606.d2

[14] Schnabl B (2013) Linking intestinal homeostasis and liver disease. Curr Opin Gastroenterol 29: 264–270. 23493073 10.1097/MOG.0b013e32835ff948 PMC4077188

[15] Blednov YA, Benavidez JM, Geil C, Perra S, Morikawa H, et al (2011) Activation of inflammatory signaling by lipopolysaccharide produces a prolonged increase of voluntary alcohol intake in mice. Brain Behav Immun 25 Suppl 1 S92–S105. 21266194 10.1016/j.bbi.2011.01.008 PMC3098320

[16] Blednov YA, Ponomarev I, Geil C, Bergeson S, Koob GF, et al (2012) Neuroimmune regulation of alcohol consumption: Behavioral validation of genes obtained from genomic studies. Addict Biol 17: 108–120. 21309947 10.1111/j.1369-1600.2010.00284.x PMC3117922

[17] Rivera CA, Bradford BU, Seabra V, Thurman RG (1998) Role of endotoxin in the hypermetabolic state after acute ethanol exposure. Am J Physiol 275: G1252–8. 9843760 10.1152/ajpgi.1998.275.6.G1252

[18] Hamann L, Alexander C, Stamme C, Zahringer U, Schumann RR (2005) Acute-phase concentrations of lipopolysaccharide (LPS)-binding protein inhibit innate immune cell activation by different LPS chemotypes via different mechanisms. Infect Immun 73: 193–200. 15618154 10.1128/IAI.73.1.193-200.2005 PMC538978

[19] Yan AW, Fouts DE, Brandl J, Starkel P, Torralba M, et al (2011) Enteric dysbiosis associated with a mouse model of alcoholic liver disease. Hepatology 53: 96–105. 21254165 10.1002/hep.24018 PMC3059122

[20] Jiang W, Lederman MM, Hunt P, Sieg SF, Haley K, et al (2009) Plasma levels of bacterial DNA correlate with immune activation and the magnitude of immune restoration in persons with antiretroviral-treated HIV infection. J Infect Dis 199: 1177–1185. 19265479 10.1086/597476 PMC2728622

[21] Caradonna L, Amati L, Magrone T, Pellegrino NM, Jirillo E, et al (2000) Enteric bacteria, lipopolysaccharides and related cytokines in inflammatory bowel disease: Biological and clinical significance. J Endotoxin Res 6: 205–214. 11052175

[22] Tang Y, Banan A, Forsyth CB, Fields JZ, Lau CK, et al (2008) Effect of alcohol on miR-212 expression in intestinal epithelial cells and its potential role in alcoholic liver disease. Alcohol Clin Exp Res 32: 355–364. 18162065 10.1111/j.1530-0277.2007.00584.x

